# The First Report of *Pennella* (Crustacea: Copepoda) Infesting *Stenella coeruleoalba* Stranded in Malta: Morphological and Genetic Analyses

**DOI:** 10.3390/ani14071107

**Published:** 2024-04-04

**Authors:** Adriana Vella, Noel Vella

**Affiliations:** Conservation Biology Research Group, Department of Biology, University of Malta, MSD 2080 Msida, Malta

**Keywords:** cetacean, DNA barcoding, mesoparasitic copepod, mitochondrial DNA, *Pennella balaenoptera*, striped dolphin

## Abstract

**Simple Summary:**

A striped dolphin was found dead in Maltese waters in July 2020, displaying a severe infestation of the mesoparasite *Pennella balaenoptera*. This parasite is the largest known mesoparasite to grow on cetaceans and the level of infestation is typically associated with the health status of its host. This incident emphasizes the importance of monitoring the presence of these parasites, to better understand the health of dolphins in the area and to contribute to conservation efforts.

**Abstract:**

Here, we document the stranding of a striped dolphin *Stenella coeruleoalba* (Meyen, 1833) (Mammalia: Delphinidae), which was found dead in Maltese waters in July 2020. The stranded dolphin exhibited a severe infestation of the mesoparasitic copepod, *Pennella balaenoptera* Koren and Danielssen, 1877 (Copepoda: Pennelidae). Parasites of this genus represent the largest known mesoparasites to infest cetaceans. Under normal circumstances, cetaceans may have a few *P. balaenoptera* individuals attached to them, but cetaceans with compromised health are more prone to heavy infestations. The identification of the parasite was accomplished through morphological and genetic analyses. This incident highlights the significance of monitoring mesoparasitic infestations, offering valuable insights into the health of cetacean populations and emphasizing the potential implications for conservation efforts in the region.

## 1. Introduction

The genus *Pennella* Oken, 1816, is composed of highly modified mesoparasitic copepods [1,2]. The life cycle of members of this genus is complex and they show variations in developmental stages and the use of intermediate hosts and definitive hosts. Typically, *Pennella* species are characterized by a brief planktonic phase, followed by a copepodid stage where they attach to an intermediate host, most likely a cephalopod [3]. Subsequently, they develop into an adult stage [4]. After mating, inseminated females parasitize the definitive host, usually a fish or a marine mammal. During this stage, the copepod’s cephalothorax including its antennary processes and holdfasts becomes deeply embedded in the host’s tissue while the posterior end, including the egg sacs and the brush-like abdominal plumes, remains outside the definitive host’s body surface [2,3,5]. During the latter stage, most of these mesoparasitic copepods become very conspicuous and, consequently, this represents the most studied stage in their complex life cycle. 

For most *Pennella* species, the specific details of their life cycle are missing, yet identification keys indicate that adult female *Pennella* species exhibit definitive host specificity, except for *P. filosa* (Linnaeus, 1758) and *P. balaenoptera* Koren and Danielssen, 1877 [2]. The former parasitizes several pelagic fish species, while the latter parasitizes different marine mammals [2,6,7,8], with the main distinguishing feature between these two species being that for *P. balaenoptera*, the definitive host is a marine mammal, usually a cetacean [2].

Currently, there are 10 to 14 species within the genus that are considered as valid or *species inquirendae* [2,9], yet mitochondrial DNA data indicate that most of these species, including *P. balaenoptera* and *P. filosa*, may be synonymous [5,7]. This genetic insight challenges the current classification and underscores the need for further molecular investigations to refine our understanding of *Pennella* species. 

Taxonomic descriptions for the majority of the *Pennella* species are biased as they have been based on specific life stages, associated with the definitive host, and limited to a few adult mesoparasitic inseminated females [2]. Consequently, sexual dimorphism, morphological variations between life stages, and phenotypic plasticity, especially that observed on the cephalothorax of these species, have led to taxonomic revisions [2]. To address these complexities, the use of standard molecular genetic techniques such as DNA barcoding [10] has become invaluable. Utilizing systems such as BOLD [11], which algorithmically clusters different operational taxonomic units (OTUs) into BIN clusters based on DNA sequences [12], allows for improvements in taxonomic identification, revisions, and biodiversity assessments [13,14,15]. This approach contributes to a more comprehensive understanding of the phylogenetic relationships between different species of *Pennella*, while assisting in better delimitation of species [5,7,16] at all life stages, allowing for improved knowledge of their complex life history. 

Accurate parasite identification is crucial not only for taxonomic purposes but also for evaluating the health of individual hosts. Studies have shown that while *Pennella* is frequently present on cetaceans [7], on healthy hosts, the normal numbers of *Pennella* individuals are typically low. Elevated infestations are often linked to underlying health conditions, such as immunosuppression related to viral infections or heavy loads of pollutants [8,17,18]. Consequently, monitoring for *Pennella* infestations can provide valuable insights into the health status of cetacean populations [19,20]. In the Mediterranean, such efforts play a crucial role in effective conservation strategies, especially in the context of various international agreements and protocols, such as ACCOBAMS [21], SPA/BD Protocol [22], CITES [23], and CMS [24], which emphasize the importance of monitoring, protecting, and conserving cetaceans [25,26,27].

The current work represents the first scientific investigation of the occurrence of this mesoparasitic copepod on a heavily parasitized cetacean in Maltese waters, offering a unique perspective from a stranding event in the central Mediterranean. 

## 2. Materials and Methods

### 2.1. Sample Collection

**Figure 1:**
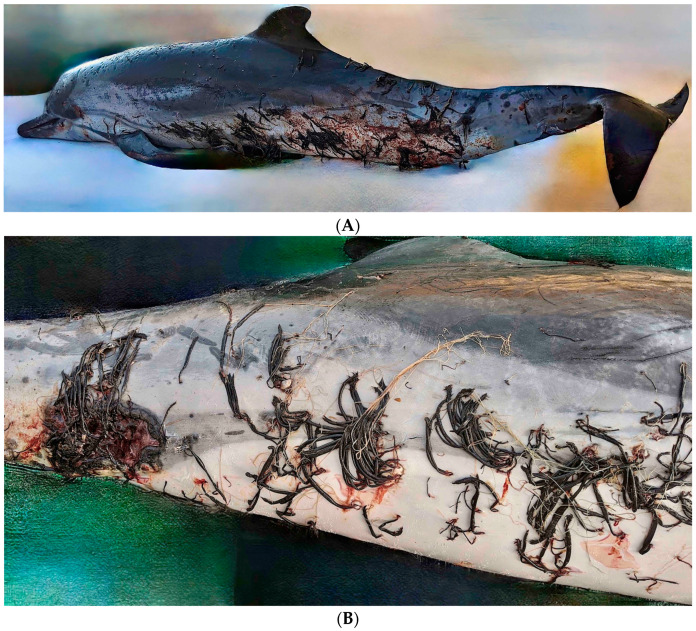
(**A**) Views of the heavily parasitized striped dolphin, *Stenella coeruleoalba*, stranded in coastal Maltese waters. (**B**) Closer view of the lower ventral anal right side of the dolphin also showing the large lesion wound close to its anus.

**Figure 2:**
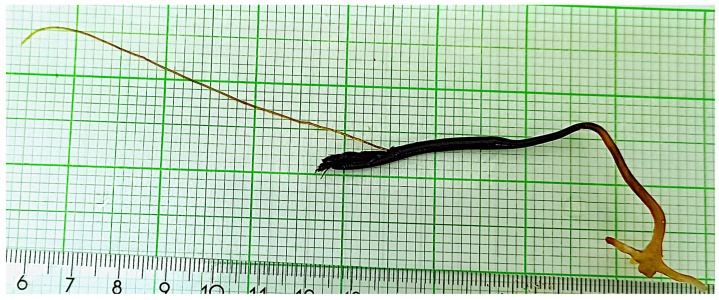
An image of a *P. balaenoptera* individual collected during this study (total length: 110 mm—measured following Suyama et al. [5]).

A dead dolphin, *Stenella coeruleoalba* (Meyen, 1833) [26], was stranded ashore on 13 July 2020, at St Julian’s, Malta (GPS coordinates: 35°55′10.4″ N 14°29′42.2″ E or 35.919556, 14.495056). During an external post-mortem examination (Figure 1), it was evident that the specimen was heavily infested with the mesoparasite *Pennella* (Figure 2). The striped dolphin was photographed and the mesoparasites visible during the post-mortem examination were counted. A few parasites including associated tissue were collected and stored in 95% ethanol until analyzed. 

The antennary processes and holdfasts of the collected parasites were examined under a low-power microscope. The morphological identification of the parasite was carried out following Hogans [2], Fraija-Fernández et al. [7], and Suyama et al. [5].

### 2.2. Genetic Analyses

The total genomic DNA was extracted from a tissue sample of the *Pennella* parasite using the GF-1 Tissue DNA Extraction Kit (Vivantis, Malaysia). The PCR amplification of the COI sequence was carried out using the genus-specific primers HijikiCOI-F (5′-GGATATTGGR ACTTTGTACTTATTAAG-3′) and HijikiCOI-R (5′-AAAAATCAAAATAAATGCTGG-3′), which prevent the amplification of cetacean DNA [5]. The amplification protocol followed Vella et al. [28]. The PCR amplification was visually confirmed on 1.5% agarose gel stained with ethidium bromide. Purified PCR products were sequenced using both the forward and reverse primers via ABI3730XL. The quality check, editing, and assembly of the complementary sequences were carried out using GeneiousR10 (http://www.geneious.com; [29]). The sequence generated during this study was deposited in GenBank under the accession number PP396156.

The sequence generated during this study was then compared against readily available data on the NCBI GenBank database via Blastn (https://blast.ncbi.nlm.nih.gov/Blast.cgi (accessed on 15 September 2023)) and the BOLD database using the Species Level Barcode Records Identification Engine (http://www.boldsystems.org (accessed on 15 September 2023); [11]).

## 3. Results

### 3.1. Morphological Analyses

This dolphin was a female of 200 cm total body length. Lengths at sexual maturity for females of this species have been reported beyond 209 cm. However, other research publishing a growth curve for Mediterranean striped dolphins shows the maximum body length to be 205 cm from around 11 years of age, while specimens at 200 cm body length would be around 7 years of age [30]. This may, however, vary according to population genetics, distribution, and general health, with unhealthy dolphins remaining smaller in size [31,32,33]. The dolphin under study had an empty stomach, except for a couple of cephalopod beaks, and its dorsal blubber thickness was 1.5 cm.

The dolphin analyzed in this study was heavily infested with 450 *Pennella balaenoptera* individuals. A higher number of *P. balaenoptera* specimens was observed on the dolphin’s lower lateral side. Notably, the dolphin must have experienced a period of physical distress due to the parasite load it was carrying; thus, it cannot be excluded that the wound on its right side may have been caused by its attempts to remove parasites by rubbing against substrates.

Although the specimen was in an advanced stage of decomposition, some other parasites were recorded. Adult nematode *Pseudaliidae* parasites were found in the lungs of the affected dolphin. Parasitic cysts (Cestoda), meanwhile, were observed mostly in the subcutaneous blubber of the dolphin’s abdominal area. Two types of Tetraphyllidean merocercoids have been widely recognized in delphinids, including *Phyllobothrium delphini* (Bosc, 1802), usually encysted in the abdominal subcutaneous blubber, and *Monorygma grimaldii* (Moniez, 1889), encysted mainly in the peritoneum of the abdominal cavity [34]. No specific investigations on the presence of viral, fungal, or bacterial pathogens or environmental contaminants were performed by the authors.

### 3.2. Genetic Analyses of the Mesoparasite

The 605 bp COI sequence produced during this study matched: 99.0% with MG701289 [host: *Grampus griseus* (Mammalia: Delphinidae)—Spain] and 98.9% with MG701287, MG701290, and MG701291 [host: *Delphinus delphis* (Mammalia: Delphinidae)—Spain], representing *P. balaenoptera* as identified in Fraija-Fernández et al. [7]; 98.5% with MG701282 [host: *Xiphias gladius* (Actinopterygii: Xiphiidae)—Spain], representing *P. filosa* as identified in Fraija-Fernández et al. [7]; and 98.8% with LC198844 and LC638618 [host: *Cololabis saira* (Actinopterygii: Scomberesocidae)—Pacific Ocean] and LC638573 [host: *Mola mola* (Actinopterygii: Molidae) Pacific Ocean], representing *Pennella* sp. as identified in Suyama et al. [5].

When the sequence was analyzed through BOLD, it clustered within BOLD:ADW8004, which contains *Pennella balaenoptera*, *P. filosa*, and *Pennella* sp., as identified in Suyama et al. [5]. This BOLD BIN is composed of 147 individuals exhibiting a maximum interspecific divergence of 3.08% (mean distance 1.06%), with its closest neighbor being BOLD:AEN6079, which also represents *Pennella* sp.

## 4. Discussion

Species delimitation becomes difficult when species exhibit morphological polymorphism that may arise due to various factors, including variations that may be associated with the host, while using identification keys that represent only a specific gender for a specific stage of a species’ complex life cycle. Consequently, the minor morphological differences noted between mature inseminated females of *P. balaenoptera* and *P. filosa* may not be enough to distinguish them reliably, leading to conflicting observations [2,7]. Instead, the main distinguishing feature may be the definitive host specificity rather than the actual parasites’ specific morphology [2]. However, while definitive host specificity may at times be an effective tool in discriminating between parasites, as some parasites tend to exhibit species-specific ecological interactions [2,35,36], in this case, the molecular data, namely those from DNA barcoding, indicated otherwise [5,7]. Our comparative genetic analyses showed that the currently studied specimen’s COI data represent a newly sequenced DNA barcode that closely matches both *P. balaenoptera* and *P. filosa*. This corroborates the outcomes from Fraija-Fernández et al.’s [7] and Suyama et al.’s [5] studies, where genetic evidence showed that these two species are probably synonymous, with *P. balaenoptera* being a junior synonym of *P. filosa*. Accordingly, mitochondrial genetics indicate that synonymy is prevalent in the genus *Pennella* [5]. 

Nonetheless, while genetics indicate that definitive host specificity cannot be used to validate a parasite species’ identification, the current work opts to keep the parasite’s name as *P. balaenoptera* rather than using *P. filosa*, a decision made since the former is considered valid by current morphological identification keys [2] and species databases [9]. 

Global records of *P. balaenoptera* indicate that inseminated females can parasitize at least 17 cetacean species [7], with Mediterranean records including *Balaenoptera acutorostrata* [37], *Balaenoptera physalus* [17,38], *Delphinus delphis* [7], *Globicephala melas* [7], *Grampus griseus* [7,18,39], *Stenella coeruleoalba* [7,20,39,40], *Tursiops truncatus* [7,39], *Phocoena phocoena* [8,41], and *Ziphius cavirostris* [7,42] as hosts. Usually, the number of parasites per host is generally low, although records for the same dolphin species indicate that as many as 344 *P. balaenoptera* parasites have previously been found on a single individual [7]. The current study of this heavily parasitized *S. coeruleoalba* surpasses the previous record for the number of parasites per specimen. Furthermore, the presence of a wound or ulceration on the lower right side suggests that either the dolphin may have engaged in rubbing itself against substrates to remove parasites or that the heavy load of this large parasite in this area together with any other underlying infection may have caused ulceration. In this regard, bottom contact behavior has been recorded in some cetacean species [19,43,44,45]. 

The fast movement of most cetaceans makes it difficult for parasites to settle, while slow-moving individuals tend to be easier hosts for colonization [40]. The high presence of *Pennella* parasites on the currently analyzed striped dolphin specimen is indicative of an immunocompromised individual that suffered from sickness-induced lethargy, possibly due to pollutants, stress, or infections [5,17]. 

The relationships and order of factors causing the dolphin’s death may be multifaceted and intricate. Just as parasites, including *Pennella balaenoptera*, may benefit from a weakened host, the parasites themselves may be carriers of infectious pathogens, such as *Brucella ceti*, which, in turn, may have long debilitating effects on the dolphin, potentially facilitating further parasite infestation. At the same time, other diseases, such as Cetacean morbillivirus (CeMV), may also coinfect or activate various other latent infections, weakening the dolphin in various life-threatening ways [32,34,46,47,48,49,50].

In the Mediterranean, CeMV and *Brucella ceti* are known to be responsible for infectious outbreaks and deaths in striped dolphins [40,46,47,51,52,53,54]. Infected individuals are or may become immunosuppressed and exhibit significant depletion of lymphoid tissue [39]. Likewise, elevated levels of polychlorinated biphenyls are also associated with immunosuppression [55]. Since sick dolphins are notably prone to opportunistic infections from various organisms, non-invasive tracking of the presence of these mesoparasites in free-living cetaceans may potentially serve as an indicator, facilitating a more comprehensive understanding of changes related to cetacean health [18]. 

## 5. Conclusions

This article represents the first scientific record of a heavily infested *S. coeruleoalba* by *P. balaenoptera* from Malta. This study has incorporated genetic analysis of the parasite because, despite being the sole *Pennella* species identified to infect marine mammals [2], the parasite species displays significant morphological variability across different life stages. Standardized identification methods, such as DNA barcoding, thus become essential in comprehending the parasite’s life cycle and contribute to our knowledge of the complexities surrounding its life stages.

Cetacean strandings and unique observations linked to these cases allow for a more in-depth consideration of the ailments these marine mammals are suffering in a degrading marine environment, which is affecting their general good health and conservation needs. However, the various causes of distress and poor health are not always easy to link to the effects observed. Nonetheless, as cetaceans play a unique and important role in the marine ecosystem and are also legally protected in most regions, flagging records, such as that in this case study, is essential to increase awareness of the status of cetacean health and measures to improve the status of their marine habitat.

## Data Availability

Mitochondrial DNA data related to the analyses conducted during this study are available on GenBank under accession number PP396156.
